# Metal Exposure, Bioaccumulation, and Toxicity Assessment in Sediments from the St. Lawrence River Before and After Remediation Using a Resuspension Technique

**DOI:** 10.3390/toxics13060432

**Published:** 2025-05-25

**Authors:** Masoumeh Javid, Catherine N. Mulligan, Marie Lefranc, Maikel Rosabal Rodriguez

**Affiliations:** 1 Department of Building, Civil and Environmental Engineering, Concordia University, 1455 de Maisonneuve Blvd. W., Montreal, QC H3G 1M8, Canada; masoumeh.javid@hotmail.com; 2 Environmental Metallomics Laboratory, Biological Sciences Department, 141 President-Kennedy Ave., Montreal, QC H2X 1Y4, Canada; lefranc.marie@courrier.uqam.ca (M.L.); rosabal.maikel@uqam.ca (M.R.R.)

**Keywords:** heavy metals, toxicity, bioaccumulation, *Hyalella azteca*, *Chironomus riparius*

## Abstract

This study, using *Hyalella azteca* and *Chironomus riparius*, evaluated the effects of exposure to heavy metal-contaminated sediments collected from the study area under three conditions: before remediation, after remediation, and suspended particulate matter (SPM). The selected toxicity tests allowed for the evaluation of biological responses across varying concentrations of heavy metals. Statistical analysis revealed no significant differences in survival or growth between sediment-exposed organisms and controls for either species. In addition, bioaccumulation of Cr, Ni, Cu, Zn, As, Cd, and Pb in both organisms was assessed and compared among the sediment conditions and the control. No statistically significant differences in tissue metal concentrations were found between organisms exposed to sediments from the study area and those in control conditions. Sequential extraction analysis indicated that a substantial proportion of metals in the sediments were bound in stable, non-bioavailable forms. These findings are consistent with the observed biological responses, as low levels of bioavailable metals corresponded with the absence of toxic effects. Together, the data confirm that the sediments, regardless of remediation stage or particle fraction, posed no significant biological risk under the conditions tested.

## 1. Introduction

Metal-contaminated sediment is a worldwide environmental problem [1]. In the aquatic environment, heavy metals have a strong affinity for particulate matter and will therefore accumulate on surface sediments. Once deposited, however, chemical and biological processes may cause substantial release of pollutants to the water column [2]. Heavy metals usually possess significant toxicity to aquatic organisms and human health through bioaccumulation in the food chain. Hence, investigating the transformation and distribution mechanisms of heavy metals in sediment becomes necessary [3]. Large-scale studies on trace element contaminants in marine [4,5], coastal [6,7], and freshwater [2,8] sediments have been reported from various countries.

The primary goal of heavy metal remediation is to reduce the potential risks these toxic substances pose to both human and environmental health [9]. While there is considerable knowledge regarding the remediation of heavy metal-contaminated soils, there is relatively limited understanding when it comes to treating contaminated sediments. Sediments often contain higher amounts of clay-size particles and organic matter compared to soils, which can affect the performance of soil-based remediation techniques when applied to sediments [10]. Choosing the most suitable remediation approach depends on various factors, including the specific site conditions, the extent of heavy metal contamination, and the regulatory standards for the metals in question. Remediation strategies are generally categorized into two main types: (1) in situ and (2) ex situ.

The use of freshwater macroinvertebrates in pollution studies has largely been restricted to biological surveys describing community changes associated with chronic and episodic exposures and to acute lethal toxicity tests in the laboratory [11]. Whether a certain technology is effective for solving the problems of soil and sediment contamination requires not only metal measurements in the sediment but also scientific tests and evaluations. For evaluation of in situ remediation effectiveness, recent studies mainly focus on three aspects: the content of pollutants, soil and sediment characteristics, and ecological risks [12]. Ecological risk is the likelihood of adverse ecological effects that are occurring or may occur as a result of exposure to one or more stressors. Stressors can be chemical (e.g., toxic or hazardous compounds), physical (e.g., habitat destruction), or biological (e.g., introduced (invasive) species, loss of prey, etc.) [13]. Ecotoxicological tests are an important part of ecological risk assessment (ERA). Due to their sensitivity and reflection of the overall toxicity, ecotoxicological tests can provide an inclusive assessment of the environmental state, which allows them to be used for evaluating the remediation effectiveness [14]. As to the ecotoxicity assessment of contaminated sediment, benthic invertebrates are used most often. Generally, benthic invertebrate species are abundant in aquatic ecosystems. Many benthic organisms are sensitive to sediment contaminants, showing a specific response to changes in pollutant type and concentration. Moreover, benthic invertebrates play important roles in the ecosystem and reflect the biodiversity of benthic communities [15]. For these reasons, benthic invertebrates are recommended species for testing the ecotoxicity of sediment [16,17]. Investigations were, therefore, initiated in several countries to select and culture benthic invertebrates which could be used for whole-sediment toxicity tests, and to develop reliable test methodologies. One of the first articles in this regard was published by Nebeker et al. from the USEPA, describing “Biological methods for determining toxicity of contaminated freshwater sediments to invertebrates”, in which the amphipod crustacean *Hyalella azteca* and the midge larva *Chironomus tentans* were the recommended test species [18]. The reasons for this selection were justified on the basis that both of these benthic invertebrates are easy to rear and test, that they remain in intimate contact with the sediment, that they exhibit high control survival, and that they are very sensitive to toxic organic chemicals [19].

The objectives of the present study were to (i) evaluate the effects of heavy metals in sediment on the survival and growth of the *Hyalella azteca* and midge *Chironomus riparius* and compare the results to the control (uncontaminated sediment), (ii) examine the survival of *H. azteca* in three sets of samples (before remediation, after remediation, and SPM), during acute exposures to heavy metals, and (iii) experimentally determine the role played by sediment in the accumulation of heavy metals by *C. riparius* larvae and *Hyalella azteca.*

## 2. Materials and Methods

### 2.1. Study Area Characteristics and Source of Contamination

The site is situated at a yacht club on the northern shore of the St. Lawrence River in Quebec, Canada. The harbor spans an area of roughly 15,000 square meters and is sheltered from wave action by two floating breakwaters and one solid breakwater. In the northwest section of the harbor, there is a boat maintenance zone used primarily for repairs and repainting during the summer months, and for boat storage in the winter. Water depths range from about 0.6 m near the docks to approximately 3 m near the floating breakwater [20].

### 2.2. Sediment Sampling

**Figure 1:**
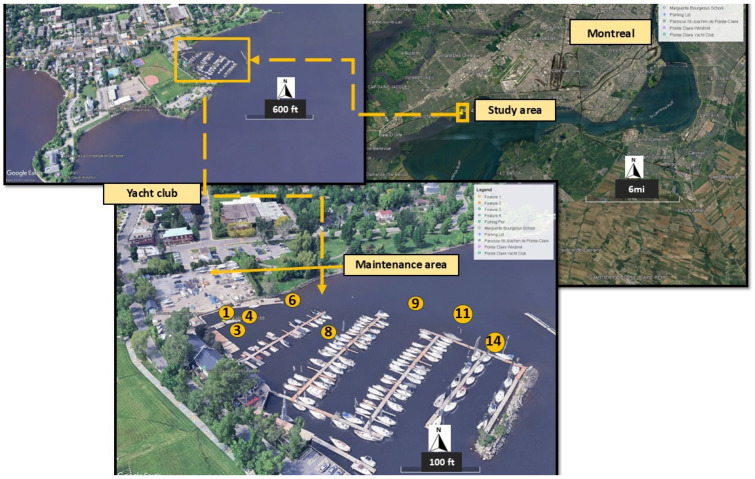
Study area with the selected numbered sampling stations, which were potential locations for dredging.

Sampling locations were chosen based on earlier research identifying pollution sources at the yacht club on the north shore of the St. Lawrence River [20]. The sampling for this study was carried out in September 2019. To assess spatial variability, eight sites were selected, and at each location, duplicate samples of surface water and sediment were collected during each sampling event (Figure 1). Sediment samples were obtained using a Birge–Ekman grab sampler from the surface down to a maximum depth of 10 cm. The collected sediments were sealed in airtight plastic bags, stored in a portable cooler at 4 °C, transported to the laboratory, and then refrigerated at the same temperature until analysis.

All plastic and glassware used in the experiments were either new or pre-cleaned by soaking in 5% (*v*/*v*) nitric acid and 2.5% (*v*/*v*) hydrochloric acid (trace metal grade) for a minimum of 8 h, followed by two rinses with deionized water produced using a Milli-Q system (18 µΩ·cm). For quality assurance, each sediment sample was analyzed alongside a blank (deionized water) and a control, in duplicate.

### 2.3. Experimental Setup of Remediation Technique

**Figure 2:**
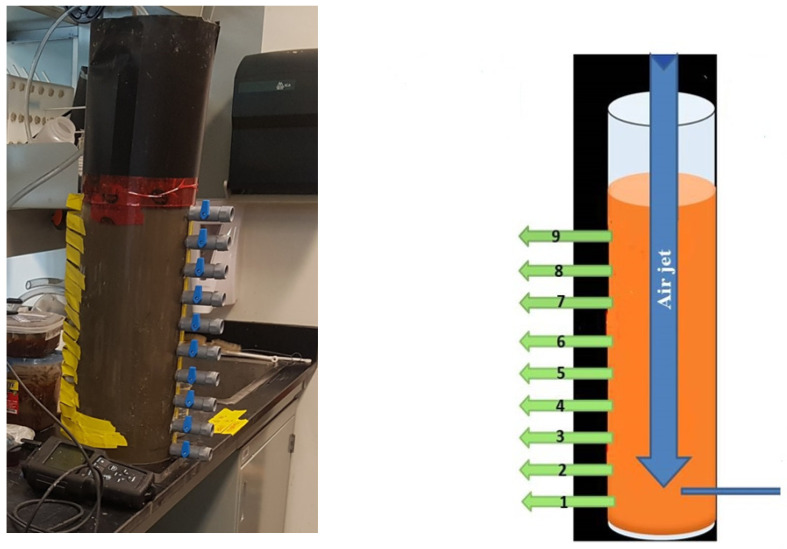
Photo and schematic of the reactor with a Plexiglas cylinder and air jet arm connected to the central air compressor in the laboratory.

The sediment resuspension system (Figure 2) consisted of three main components. The first component involved resuspension and aeration using an air jet connected to the lab’s central compressed air supply. The setup featured a vertical cylindrical reactor measuring 20 cm in internal diameter and 50 cm in height. Several outlet holes were positioned at various heights along the cylinder wall: hole 1 (1.5 cm), hole 2 (6.5 cm), hole 3 (11.5 cm), hole 4 (16 cm), hole 5 (21 cm), hole 6 (26 cm), hole 7 (31 cm), hole 8 (35.5 cm), and hole 9 (40.5 cm) (Figure 2). During each experiment, sediment samples were first homogenized and then mixed with tap water in a 1:10 (*V*/*V*) ratio inside the reactor, with the total mixture reaching a height of 40 cm. Tap water was used because its properties closely resemble those of the river’s freshwater. A plastic lid was placed on the reactor to prevent splashing during the aeration process, as the turbulence created by the air jet was quite intense.

**Figure 3:**
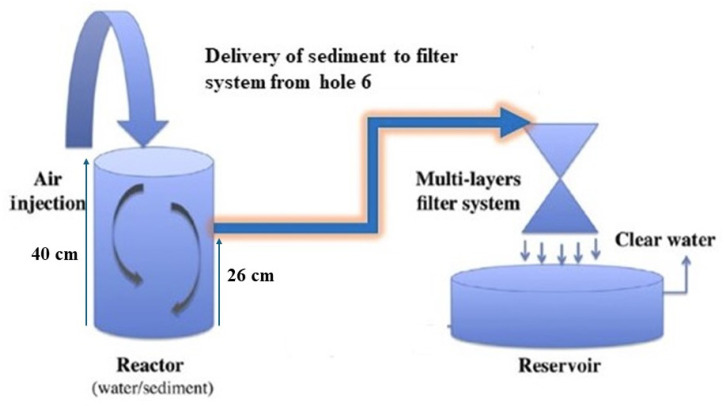
Schematic of the resuspension process in the laboratory.

In the second phase, aeration was stopped after two hours, allowing the heavier particles to begin settling. Within approximately 15 min, sand and coarse silt had largely settled out. The remaining slurry—containing suspended particulate matter (SPM) and some insoluble organic material—was then extracted from hole 6 and transferred to the filtration system (Figure 3). The reactor can also be adapted for field use as a self-contained unit. 

### 2.4. Analytical Parameters

#### 2.4.1. Grain Size

Sediment grain size was analyzed using a laser scattering particle size analyzer (HORIBA, LA-950V2, Horiba Canada Inc., Burlington, ON, Canada). Results were reported as D50, indicating the particle diameter below which 50% of the sample falls, along with the proportions of clay, silt, and sand in each sample are shown in Supplementary Materials (Tables S1 and S2).

#### 2.4.2. Total Concentration of Heavy Metals

The total concentrations of metals—cadmium (Cd), copper (Cu), zinc (Zn), chromium (Cr), nickel (Ni), arsenic (As), and lead (Pb)—in sediment samples were measured using acid digestion in accordance with the EPA 3050B method [20]. This involved digesting 1 g of dried sediment with multiple additions of 5 mL of nitric acid (HNO_3_, 70%, trace metal grade) and 9 mL of hydrogen peroxide (H_2_O_2_, 30%), followed by hydrochloric acid (HCl, 35%) during the final stage of digestion [20]. After digestion, the samples were refrigerated at approximately 4 °C until analysis. All plastic and glassware used in the procedure were either new or pre-treated by soaking in 5% (*v*/*v*) nitric acid and 2.5% (*v*/*v*) hydrochloric acid (trace metal grade) for a minimum of 8 h, then rinsed twice with deionized water produced by a Milli-Q system (18 µΩ·cm). For quality assurance, each sediment sample was tested alongside a blank (deionized water), a control, and was analyzed in duplicate. Elemental analysis was conducted using an ICP-MS equipped with a quadrupole mass analyzer (Agilent 7700x, Agilent Technologies, Mississauga, ON, Canada) to measure metal concentrations. Analyses of sediment and SPM metal concentrations are found in the Supplementary Materials (Tables S3–S5).

#### 2.4.3. Sequential Extraction Method

Sequential extraction analysis was performed to assess how contaminants in the sediment samples were partitioned among different chemical forms. The method, from Yong et al. [21], was used, and it classifies the contaminants into five distinct fractions: (1) exchangeable, (2) associated with carbonates, (3) bound to iron and manganese oxides, (4) bound to organic matter, and (5) residual. Metal analyses were performed by ICP-MS for each fraction.

#### 2.4.4. Toxicity Tests

Aquatic toxicity testing was conducted to assess and track the harmful effects of individual contaminants or complex mixtures on native aquatic organisms in both the overlying water column and sediment. Survival and growth tests were performed using the freshwater species *Chironomus riparius* and the amphipod *Hyalella azteca*, applied to both the original (untreated) sediments and those that had undergone decontamination.

##### 10 Days Survival/Growth Test of *Chironomus riparius*

The test involving the midge *Chironomus riparius* was conducted according to the standardized method established by Environment Canada [22]. The chironomids used in the experiment were sourced from in-house laboratory cultures maintained in accordance with OECD [23] and Environment Canada [24] guidelines. The test was carried out using first-instar larvae that were less than two days old. One day prior to the test, sediment samples were homogenized and placed in 500 mL plastic beakers to a height of 1.75 cm. Each beaker was then filled with 325 mL of reconstituted water, resulting in a water column of 7 cm. This created a sediment-to-water height ratio of 1:4, as recommended by OECD guideline 218. The beakers were kept in a temperature-controlled room at 23 °C, covered with lids, and aerated using glass Pasteur pipettes.

Each treatment was tested in four replicates, while five replicates were prepared using reference sediment. At the start of the experiment, ten larvae were introduced into each beaker. The survival and growth test lasted for 10 days under static conditions at a temperature of 23 ± 1 °C. Aeration was initiated 24 h before introducing the larvae and continued throughout the experiment at a rate of 2–3 bubbles per second. A 16:8 h light-to-dark cycle was maintained at an intensity of 500–1000 lux. Larvae were fed daily with 0.6 mg of TetraMin (delivered as 1.5 mL of a 4.0 mg/mL suspension) per beaker. Water quality parameters—including temperature, pH, dissolved oxygen, conductivity, nitrate (NO₃^−^), nitrite (NO₂^−^), and ammonium (NH₄^+^)—were measured at both the beginning and end of the test period, while temperature and dissolved oxygen were monitored three times per week. All parameters respected the allowed range established by Environment Canada [22].

##### 14 Days Survival/Growth Test of *Hyalella azteca* Tests

The test using the amphipod *Hyalella azteca* was carried out following the standardized protocol by Environment Canada [24]. Juvenile amphipods, aged between 2 and 9 days (with a maximum age difference of 3 days), were sourced from in-house lab cultures maintained according to standard procedures [24]. One day prior to the experiment, sediment samples were homogenized and added to 500 mL plastic beakers to a depth of 1.75 cm. Each beaker was then filled with 325 mL of reconstituted water, creating a water column of 7 cm and a sediment-to-water height ratio of 1:4. The beakers were kept in a temperature-controlled room at 23 °C, covered with lids, and aerated using glass Pasteur pipettes.

Each treatment included four replicates, while five replicates were prepared using reference sediment. At the start of the test, ten juveniles were introduced into each beaker. The test was conducted over a 14-day period under static conditions (with no water changes except to offset evaporation) at a temperature of 23 ± 1 °C and was used to assess both survival and growth.

Aeration was initiated 24 h prior to the introduction of test organisms and maintained throughout the entire experiment at a rate of 2–3 bubbles per second. A 16 h light and 8 h dark photoperiod was used, with light intensity set between 500 and 1000 lux. Amphipods were fed three times per week with 6.3 mg of TetraMin^®^ per beaker. Water quality parameters—including temperature, pH, dissolved oxygen, conductivity, nitrate (NO₃^−^), nitrite (NO₂^−^), and ammonium (NH₄^+^)—were measured at both the beginning and end of the exposure period. Additionally, temperature and dissolved oxygen were monitored three times a week for all treatments, while pH and ammonia were checked three times weekly in the reference sediment group.

#### 2.4.5. Growth Parameters

Larvae (both *Hyalella azteca* and *Chironomus riparius*) grow exponentially (logarithmically) with time, so that, for each treatment, the mean specific growth rate of the larvae (SGR, in day^−1^) was calculated according to [25,26]:

(1) $$SGR=\frac{\ln(L_{final})-ln(L_{initial})}{t}$$

where L_initial_ and L_final_ are, respectively, the initial and final average larvae length and t is the exposure period (i.e., 14 days). The SGR was then normalized by the SGR of the larvae exposed in the controls (SGR_control_) to obtain relative growth rate (RGR, in percent):

(2) $$RGR=\frac{SGR}{{SGR}_{control}}\times 100$$

#### 2.4.6. Length Measurements

Organism length was measured using a calibrated image analysis system or a digitizing microscope, which allowed for magnification and precise measurement of preserved specimens. Body size was recorded to the nearest 0.001 mm from digital images using ImageJ software (version 1.54f).

#### 2.4.7. Bioaccumulation Analysis

After 48 h of depuration at 23 °C ± 1 °C with reconstituted water, larvae were rinsed with 10 mM EDTA (free acid, Bio Basic, Montreal, QC, Canada) three times during 2 min of agitation according to [27,28] in order to remove adsorbed metals. Samples were put at −80 °C before freeze drying for 24 h and kept until analysis. The samples were digested in order to be able to analyze their heavy metal concentrations according to the method of Rosabal et al. [29] with some modifications. To start digestion, nitric acid (70%, *v*/*v*, Optima grade, Fisher Scientific, Whitby, ON, Canada) was added at 2% of the final volume (check your volume in µL), then the samples were rested for 24 h at room temperature. The tubes were heated to 65 °C for 4 h. After heating, hydrogen peroxide (30%, *v*/*v*; Optima grade, Fisher Scientific, Whitby, ON, Canada) was added to 2% of the final volume, then the samples rested for another 24 h at room temperature. Finally, the samples were transferred to 15 mL metal-free tubes, and ultrapure water was added to reach a final volume of 5 mL. To ensure digestion quality, reference material ERM-CE278K (Certified reference mussel tissue) and blanks were also digested using the same method in six replicates each to determine the recovery percentage.

#### 2.4.8. Quality Assurance

All reagents used in the experiments were of analytical grade. Each sample was processed in triplicate. Plastic and glassware were either new or pre-treated by soaking in 5% (*v*/*v*) nitric acid and 2.5% (*v*/*v*) hydrochloric acid (trace metal grade) for a minimum of 8 h, followed by two rinses with deionized water produced by a Milli-Q system (18 µΩ·cm). For quality assurance, all sediment and larval samples were tested alongside blanks, controls, and duplicates. The detection limits for metals were 0.007 ppb for Cr, 0.002 ppb for Ni, 0.03 ppb for Cu, 0.1 ppb for Zn, 0.01 ppb for As, 0.002 ppb for Cd, and 0.009 ppb for Pb. Recovery efficiencies were assessed using the certified reference material EnviroMat contaminated soil, following the same analytical procedure as for field samples. The average recovery rate for the heavy metals was 104.7%. Additionally, digestion quality was validated using six replicates of the reference material ERM-CE278K (certified mussel tissue), which yielded an average recovery of 91.8% for the same metals.

### 2.5. Data Analysis

Statistical analysis was conducted to assess significant differences in survival and growth among treatments. The Shapiro–Wilk test and Bartlett’s test were applied to evaluate the assumptions of normality and homogeneity of variances, respectively. Only the sediment data met these assumptions and were analyzed using one-way ANOVA followed by Tukey’s post hoc test. The remaining datasets, which did not meet the assumptions, were evaluated using the Kruskal–Wallis test followed by Dunn’s test. All statistical analyses were carried out using RStudio (version 2023.12.0), with a significance level set at *p* < 0.05.

To explore the relationship between metal accumulation in *Hyalella azteca* and *Chironomus riparius* and the fractionation of metals in sediments, both Pearson and Spearman correlation analyses were performed. The statistical significance of these correlations was assessed using Pearson’s correlation coefficient at 95% (*p* < 0.05) and 99% (*p* < 0.01) confidence levels.

## 3. Results and Discussion

### 3.1. Quality of the Sediment Before Resuspension

**Figure 4:**
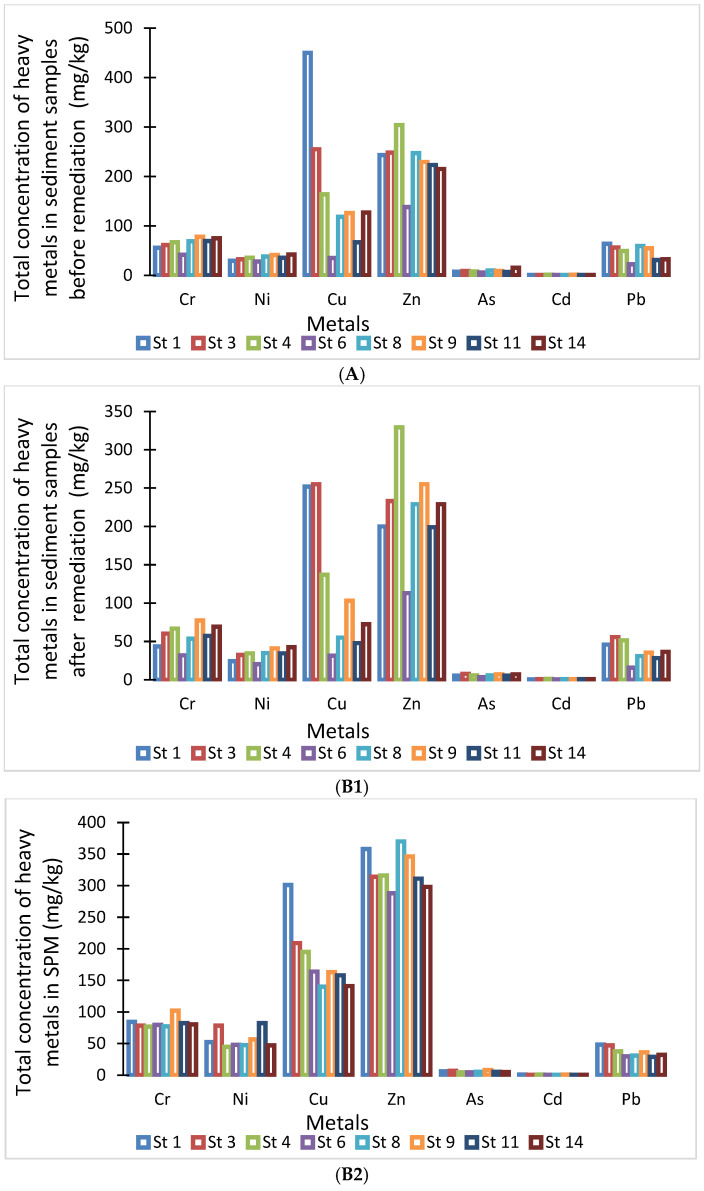
Total concentration of heavy metals in (**A**) before the remediation test (mg/kg), in (**B1**) after the resuspension test (mg/kg), and (**B2**) in SPMs (mg/kg).

Figure 4A presents the concentrations of heavy metals (Cr, Pb, Cd, As, Cu, Zn, and Ni) measured in sediment samples before the remediation test. Notably, levels of Cr, Cu, and Zn frequently surpassed guideline values across several stations. As a result, the sediments can be classified as contaminated and warrant proper management. Continued monitoring and further health risk assessments are recommended, especially for metals exceeding regulatory thresholds.

Stations 1 through 4 were identified as the most polluted, which aligns with expectations, as these sites receive runoff from the boat maintenance area, an area commonly associated with antifouling paint residues. The findings indicate that Zn, Cr, and Cu are the primary pollutants present in the sediment. Given that copper, zinc, cadmium, and lead are common components in many antifouling paint formulations, the elevated concentrations of Zn, Cr, Ni, and Cu are likely linked to these coatings.

The extensive use of antifouling paints has led to significant pollution in aquatic environments. During boat maintenance activities such as repainting and repairs, particles from antifouling paints (APPs) are released and carried into waterways via surface runoff, eventually accumulating in harbor sediments. These APP particles are a major source of inorganic, non-degradable biocidal compounds in sediment. Historically, antifouling paints included toxic substances like copper and tributyltin (TBT). However, with the recent ban on triorganotin compounds such as TBT, modern antifouling paints now primarily rely on copper(I)-based biocides, often combined with zinc oxide as a booster. Additionally, newer paint formulations may include various additives and non-biocidal pigments containing heavy metals such as lead antimonates [Pb(SbO_3_)_2_], lead chromates, and cadmium yellow.

### 3.2. Resuspension Effects on the Quality of Contaminated Sediment

Figure 4(B1) presents the total concentrations of heavy metals in sediment samples following two hours of resuspension and the subsequent removal of suspended particulate matter (SPM) from the reactor. The results demonstrate that the resuspension technique effectively reduced heavy metal concentrations across all samples. Initially, five metals exceeded the OEL at seven stations; after treatment, this was reduced to four metals across the same number of stations. Notably, at station 3, the copper concentration dropped from above the PEL to below the OEL, indicating a significant reduction. Figure 4(B2) provides the total concentrations of heavy metals found in the suspended particulate matter (SPM). The particle size of SPMs across all sediment samples ranged between 0.17 and 9.66 microns, corresponding to clay and silt-sized particles. Overall, SPMs were found to be more contaminated than the bulk sediment, consistent with findings from previous research [20].

In a recent study by Javid and Mulligan [30], the total concentrations of heavy metals (As, Cd, Cr, Cu, Ni, Pb, and Zn) were compared across three sediment types: before remediation, after remediation, and suspended particulate matter (SPM). Prior to remediation, the concentrations of several metals, particularly Cu, Cr, and Zn, exceeded the occasional effect levels, indicating significant contamination at multiple sampling stations. Following remediation via the resuspension technique, a notable reduction in metal concentrations was observed. Cu showed the highest removal efficiency at 32.4%. SPM, collected post-resuspension, showed elevated concentrations of metals in the more labile fractions, suggesting effective separation of mobile contaminants from the bulk sediment.

### 3.3. Survival and Growth (RGR) of Amphipod Hyalella azteca in Before-Remediation Sediment Samples

#### 3.3.1. Survival of Amphipod *Hyalella azteca* in Before-Remediation Sediment Samples

**Figure 5:**
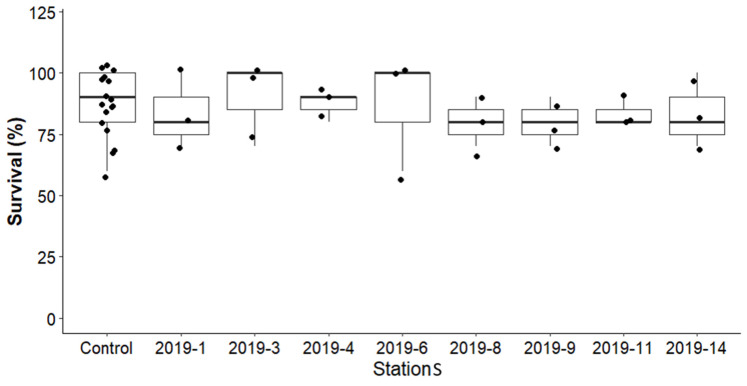
Comparison of the survival percentage of *Hyalella azteca* between the controls of the different tests (batch) with before-remediation samples at various stations.

The toxicity test results from samples collected before remediation were compared to those obtained after remediation and from suspended particulate matter (SPM), each with varying levels of heavy metal contamination. Figure 5 presents the survival rate of *H. azteca* in sediment samples collected prior to remediation. The average survival in the control group during the 14-day exposure period met the test validity criteria (≥80%) outlined by Environment Canada [24]. Survival rates across all stations did not significantly differ from the control (Figure 5), and there was no statistically significant correlation between total heavy metal concentrations and mortality (*p* > 0.05). Amphipod survival exceeded 80% at all stations, indicating that sediments in this area did not exhibit sub-chronic toxicity to *H. azteca*.

#### 3.3.2. Growth of Amphipod *Hyalella azteca* in Before-Remediation Sediment Samples

**Figure 6:**
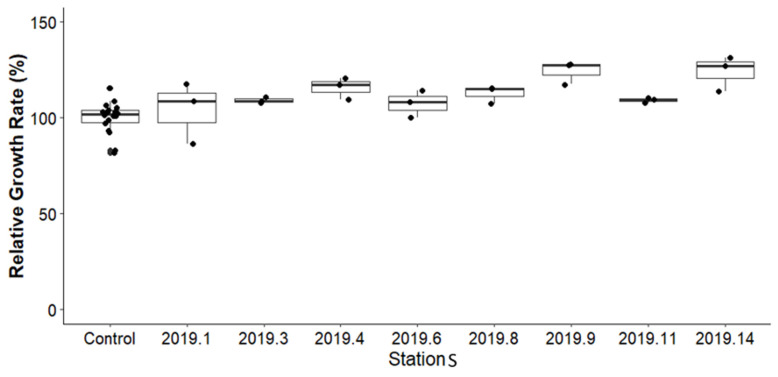
Comparison of the RGR percentage of *Hyalella azteca* between the controls of the different tests (batch) and stations for before remediation samples at various stations.

The OECD guidelines recommend using the relative growth rate (RGR) as an indicator for assessing chemical toxicity [31]. RGR was calculated for each sediment treatment group using Equation (2). Figure 6 presents the average growth values of *H. azteca* from eight stations sampled, compared against the control group. Statistical analysis indicated no significant differences in RGR between sediment samples from the study area and the control. Control RGR values ranged from 100% to 100.7%, while the sediment samples ranged from 94% to 132%.

In all stations, *H. azteca* showed a greater average body size compared to their respective control, suggesting no direct correlation between total metal concentrations and the observed growth increase. One possible explanation is that metal concentrations were not high enough to hinder growth. Additionally, differences in sediment composition may have contributed. The control sediment, being sandy and containing only 1% organic matter, might lack essential nutrients necessary for optimal amphipod development. Despite equal food inputs across treatments, the higher organic matter content in sediments from the river stations may have enhanced growth conditions for *H. azteca* [32]. Overall, there were no significant differences in RGR or survival between the control and sediment samples prior to remediation.

### 3.4. Survival and Growth (RGR and Weight) of Amphipod Hyalella azteca in After Remediation Sediment Samples

#### 3.4.1. Survival of Amphipod *Hyalella azteca* in After Remediation Sediment Samples

This study assessed sediments from four stations to evaluate the impact of heavy metals on *Hyalella azteca* survival following remediation. Results are shown in the Supplementary Materials (Table S1). In the 14-day exposure period, the mean survival rate in the control group met the Environment Canada [24] threshold for test validity (>80%). Survival rates across all stations after remediation did not significantly differ from the control, and no correlation was found between heavy metal concentrations and mortality (*p* > 0.05). Survival rates of ≥80% were observed at all monitored stations, suggesting that post-remediation sediments did not exhibit acute toxicity toward *H. azteca*. Results are shown in the Supplementary Materials (Figure S1).

A key aim of this study was to compare *H. azteca* survival across three sediment types: pre-remediation, post-remediation, and suspended particulate matter (SPM), during short-term exposure to heavy metals. While most metal concentrations in post-remediation sediments were reduced, with the exception of Cr and Cu at site 9, survival outcomes remained consistent. This indicates that the reduced metal levels did not influence amphipod mortality, and no toxicity was observed at any concentration level after remediation.

#### 3.4.2. Growth of Amphipod *Hyalella azteca* in After Remediation Sediment Samples

*Hyalella azteca*, a freshwater amphipod, is commonly used in 14-day sediment toxicity tests to assess survival. However, growth is also considered a valuable endpoint, providing additional insight into sublethal effects [33]. Assessing sublethal responses like growth can often serve as a more sensitive biological indicator and a more reliable predictor of toxicity in contaminated sediments compared to survival alone [34,35].

The post-remediation sediment samples had a noticeable impact on amphipod growth, measured as the relative growth rate (RGR). Sediments collected from four stations were chosen to evaluate these growth effects. Results are shown in the Supplementary Materials (Table S2).

Relative growth rates (RGRs) were determined for each sediment treatment group using Equation (2). The mean growth values of *Hyalella azteca* from post-remediation sediment samples collected at four stations were analyzed and compared to those of the control group. Statistical analysis showed no significant difference in mean RGR between the sediment samples and the control. While control samples had an average RGR of 100%, the sediment samples showed higher values ranging from 110% to 133%. An overall increase in the average body size of the amphipods was observed across all stations compared to their respective controls. Results are shown in the Supplementary Materials (Figure S2).

### 3.5. Survival and Growth (RGR and Weight) of Amphipod Hyalella azteca in SPM Sediment Samples

By assessing the chronic toxicity of heavy metals, linkages between bioaccumulation and growth and survival during chronic exposures were evaluated.

#### 3.5.1. Survival Amphipod *Hyalella azteca* in SPM Sediment

Survival outcomes for SPM were evaluated for stations 1, 3, 4, and 14 over a 14-day exposure period. Control groups consistently showed survival rates of ≥80%, meeting the standard criteria. Comparisons of survival data indicated no significant differences between control and SPM samples, aligning with results from both pre- and post-remediation sediments. There was no observed correlation between heavy metal concentrations in SPM and organism survival, and mortality remained low across all treatments. The average mortality rate was 6% in controls and 10% across all SPM treatments. These findings suggest that *Hyalella azteca* maintained high survival rates under all conditions—before remediation, after remediation, and in SPM—despite higher total metal concentrations in SPM. This indicates that the amphipods were not adversely affected by the contaminated sediment. Results are shown in the Supplementary Materials (Figure S3).

#### 3.5.2. Growth of Amphipod *Hyalella azteca* in SPM Sediment

The growth rate was quantified by determining the RGR. Heavy metal concentrations did not cause a significant decrease in growth rate compared with the control (*p* < 0.05). In general, inhibition of growth was not seen in *H. azteca* across all tests. Results are shown in the Supplementary Materials (Figure S4).

The average growth parameters of *Hyalella azteca* from four SPM sampling stations were assessed and compared to control values. Statistical analysis indicated no significant differences in the mean relative growth rate (RGR) between SPM sediment samples and the control group. Control RGR values averaged 100%, while SPM sediment samples ranged from 90% to 101%. This study found no variation in sensitivity between the two biological endpoints—survival and growth rate—when assessing the effects of heavy metals.

In research conducted by Borrely et al. [36], exposure of *Hyalella azteca* larvae to sediment concentrations of 21.2 mg/kg arsenic, 71.3 mg/kg chromium, and 31.11 mg/kg lead resulted in increased mortality. These levels, except for arsenic, were not substantially higher than those found in the river sediments of the present study, suggesting that lower concentrations are unlikely to produce toxic effects. Similarly, a study by Liber et al. [37] reported a 50% decline in *H. azteca* survival when exposed to 521 mg/kg nickel and 532 mg/kg arsenic—concentrations that are notably higher than those measured in the St. Lawrence River sediments (except for arsenic and nickel). These findings support the notion that the absence of toxic effects in the present study is consistent with the lower contamination levels observed.

### 3.6. Survival and Growth (RGR and Weight) of Chironomus riparius for Before-Remediation Sediment Samples

To evaluate the survival effects of various chemical concentrations (heavy metals), test organisms were exposed to different sediment samples (before remediation, after remediation, and SPM). Survival data were analyzed statistically, and the mean survival percentage of larvae was calculated for each treatment. The average survival rate in controls was 88.2%, which exceeds the 70% survival threshold set by Environment Canada [25] for a valid replicate.

#### 3.6.1. Survival of *Chironomus riparius* for Before-Remediation Sediment Samples

**Figure 7:**
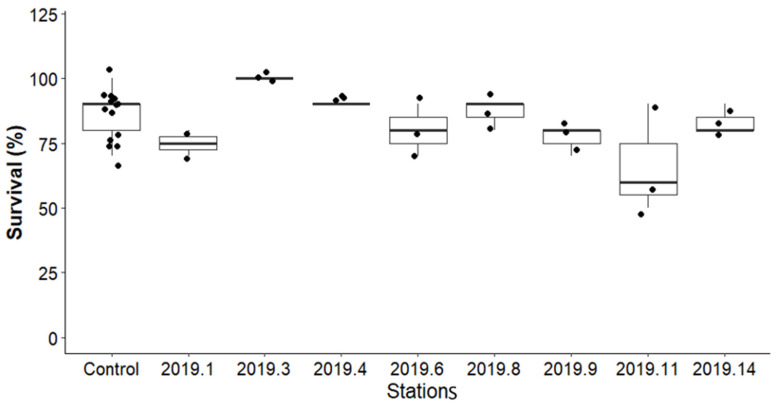
Comparison of the survival percentage of *Chironomus riparius* between the controls and before remediation samples at various stations.

Survival results are shown in Figure 7. The 10-day exposure period resulted in consistent survival rates in controls (≥70%). The mean mortality rate in the control was 11.8%, while the mean mortality across all sediment treatments was 19.7%. No significant difference in survival percentage was observed in the sediment samples before remediation compared to the control, regardless of the heavy metal concentrations at various stations. However, organisms exposed to sediment from station 11 showed reduced survival rates of 66.66% at both concentrations.

#### 3.6.2. Growth of *Chironomus riparius* for Before-Remediation Sediment Samples

**Figure 8:**
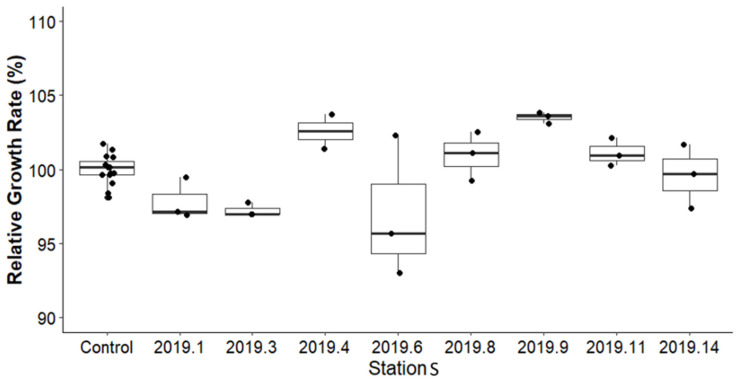
Comparison of the RGR percentage of Chironomus riparius between the controls and before remediation samples at various stations.

RGR was calculated for each sediment sample treatment group using Equation (2). The average growth parameters of *Chironomus riparius* from eight stations for sediment before remediation are shown in Figure 8 and compared with control values. Statistical analysis indicated no significant difference in the mean RGR between the sediment samples from the study area and the controls. The average RGR for controls was 100%, while the RGR for sediment samples ranged from 96% to 106%. In some stations, an increase in the average size of *Chironomus riparius* was noted compared to their respective control.

### 3.7. Survival and Growth (RGR) of Chironomus riparius in After Remediation Sediment Samples

A 10-day exposure period resulted in consistent survival rates in the controls (≥70%). In the before-remediation sediment, no significant difference in survival percentage was observed at any of the heavy metal concentrations across different stations. However, organisms exposed to sediment from station 1 showed reduced survival rates (56.7% and 56.7%, 40% and 66.7%, respectively). Results are shown in the Supplementary Materials (Figure S5).

Relative growth rate (RGR) was calculated for each sediment sample treatment group using Equation (2). The average growth parameters of *Chironomus riparius* from four stations after remediation were analyzed and compared with control values. Statistical analysis showed that the mean RGR did not significantly differ between sediment samples and controls. The control group had an average RGR of 100%, while sediment samples ranged from 98% to 103%. Results are shown in the Supplementary Materials (Figure S6).

In a study by [38], larvae from a different chironomid species, *Chironomus tentans*, were exposed to concentrations of 5 μg/g Cd, 10 μg/g Cu, 70 μg/g Pb, and 300 μg/g Zn, which resulted in increased larval mortality. These concentrations were much higher than those found in the river sediments, except for Cu. Consequently, it seems reasonable to expect no toxic effects at lower concentrations. However, the study used a different species, a 14-day exposure period (compared to the 10-day period in this study), and synthetic sediment with added metals, which may have influenced the bioavailability and mobility of the contaminants.

Therefore, the actual contaminated sediments used in this study could lower metal bioavailability, which, in turn, reduces their toxicity. Several factors, such as the concentration of AVS (acid volatile sulfide), dissolved organic carbon, and the interactions between different metals, can influence this process. In a study by Arambourou [39], the effects of metal-contaminated sediments on *Chironomus riparius* larvae were evaluated. They observed a decrease in larval mass when exposed to concentrations of 53 mg/kg As, 27.8 mg/kg Cd, 40.7 mg/kg Cr, 165 mg/kg Cu, 1.87 mg/kg Hg, 45.7 mg/kg Ni, 1905 mg/kg Pb, and 11,221 mg/kg Zn. These concentrations are much higher than those found in the St. Lawrence River, except for Cr, Cu, and Ni. Additionally, the larvae in this study were exposed for 15 days, five days longer than in our study, which could have influenced the results.

Given this, it is expected that the results from our study are inconclusive, especially since other studies that reported reductions in weight, size, or survival of larvae of this species or other chironomids were conducted with higher metal concentrations or longer exposure periods.

### 3.8. Impact of Geochemical Fractions of Metals on Their Bioaccumulation

It is widely recognized that the sequential extraction technique helps predict the potential impacts of metals on biota in aquatic environments. Understanding the chemical speciation of trace metals is crucial for assessing their reactivity, mobility, and toxicity in aquatic systems [40]. This study performed a correlation analysis to explore the relationship between the bioaccumulation patterns of heavy metals in test organisms (*Chironomus riparius* and *Hyalella azteca*) and the partitioning of metals in sediments, as presented in Table 1 and Table 2.

Table 1 displays the correlation between metal levels in *Chironomus riparius* tissues and the geochemical fractions of sediment. The residual (F5) fraction in sediment showed strong positive associations with Pb concentrations in *Chironomus riparius* (*p* < 0.05), indicating that residual forms of Pb enhance its bioaccumulation in the organism. Total As concentrations in sediment showed very strong positive correlations with As concentrations in *Chironomus riparius* (*p* < 0.01). In contrast, there was a significant negative correlation between *Chironomus riparius* and the exchangeable fraction (F1) for Cr, Cu, and Zn in sediment (*p* < 0.01). Similarly, a negative correlation was observed between *Chironomus riparius* and the acid reducible fraction (F2) for Ni and As in sediment (*p* < 0.05 for Ni and *p* < 0.01 for As). The acid-reducible fraction is known to be a major sink for heavy metals in aquatic environments [41]. For Cd and Pb, most correlations were negative. Generally, the concentrations of metals in fraction I (exchangeable) correlated negatively with bioaccumulation, suggesting that the metals in this fraction did not significantly influence their ecotoxicity to the test organisms. Studies have not confirmed metals in the exchangeable fraction (I) as bioavailable or toxic to organisms, as most correlations between metal concentration in fraction I and organism response were negative.

The relatively low sensitivity of *C. riparius* larvae to the bioavailable fraction of metals in the sediment in this study was anticipated, as chironomid larvae are benthic organisms (i.e., they live in tubes within the sediment) and primarily feed on organic matter that is deposited or mixed with the sediment. As deposit feeders, the material they ingest plays a crucial role in both the bioaccumulation and toxicity of metals to *C. riparius* larvae [42]. The exposure route to contaminants in chironomids depends on both the contaminant type and the test conditions. Chironomids burrow in the upper layers of sediment but remain in close contact with surface water, where they obtain food and oxygen. Water flowing over their gills can be a significant source of contamination. Additionally, chironomids consume food particles that they either suck into their sheath or gather from the sediment or surface. In doing so, they may also ingest similarly sized particles, such as silt, which could lead to contamination if those particles are tainted. While chironomids can sometimes selectively choose non-contaminated particles, they can still become poisoned from ingesting contaminated food or non-food particles. Generally, chironomids are not the most sensitive species to metal contamination, but they serve as effective models for studying the toxicity of organic compounds, which may affect them more severely than other species like *D. magna*, *H. azteca*, and *P. promelas* [43].

The exposure of chironomid larvae to heavy metals in the water column is influenced by several factors. Although these larvae reside in the surface sediment layer and may come into contact with waterborne contaminants, their tubes provide some shielding from these contaminants. The extent of this protection depends on the type of particles used to construct the tube [42]. Halpern et al. [44] discovered that silt tubes offered better protection to *Chironomus luridus* against copper exposure than sand tubes. Given that the sediment in the present study consists of 35–50% sand and 46–67% silt and clay, the tubes of *C. riparius* larvae likely provided significant protection against heavy metals. As a result, the larvae were relatively shielded from metals dissolved in water, which are in a more bioavailable form. This could explain, at least partially, the negative but significant correlation between the bioavailable fraction of metals and bioaccumulation, as well as the good survival rate of *C. riparius* in the sediment samples. Studies by Amiard et al. [45] have shown that chironomids are better able to regulate concentrations of essential metals (such as Cu and Zn) than nonessential metals (such as Cd and Pb).

Faria et al. [42] suggested that the primary toxicity to *C. riparius* larvae is caused by metals that enter the organism through the ingestion of inorganic sediment particles. Their findings show that the bioavailability of heavy metals to the larvae is heavily influenced by the ingestion of these inorganic particles. This means that the particle size of the sediment can significantly impact the bioavailability and toxicity of heavy metals, as the size of particles larvae can ingest is determined by the width of their mentum [42]. Since inorganic sediment particles are consumed along with natural or added food (organic matter), the bioavailability and toxicity of heavy metals in sediment, related to the inorganic fraction, may also depend on the quantity and quality of the food. Both types of food can directly affect the amount of inorganic sediment particles ingested (by altering the balance of organic and inorganic particles consumed) and/or indirectly (by influencing the nutrient content of the food, which, in turn, impacts the ingestion rate) [46].

*Hyalella azteca* is highly sensitive to metal toxicity [47]. Table 2 illustrates the correlation between metal concentrations in *Hyalella azteca* tissues and the geochemical fractions. It reveals a positive correlation between the F2 fraction of Cr, Ni, Cu, and As with their respective concentrations in *Hyalella azteca*. The F3 fraction of Cr, Cu, and As also shows a positive correlation with their concentrations in *Hyalella azteca*. This indicates that the influence of Cr, Cu, Ni, and As, associated with the labile fractions (F1 + F2 + F3) of the study area sediments, is significant in terms of bioaccumulation. Previous studies have similarly suggested a strong relationship between metal bioaccumulation and the more mobile fractions (F1 and F2) in sediments [48,49,50,51].

Ecotoxicology has heavily focused on understanding how organisms are exposed to contaminants and how exposure influences bioaccumulation and resulting toxicity [52]. One theory suggests that, at lower exposure concentrations, organisms regulate metal excretion at or above metal uptake, detoxifying accumulated metals by sequestering them into nonbiologically active pools, such as metallothionein-like proteins or metal-rich granules. However, at high metal exposure levels, organisms may take up metals at a rate that exceeds their excretion, or the nonbiologically active pools may reach saturation, causing metals to spill into biologically active pools and leading to harmful effects [53].

*Hyalella azteca* is frequently used in standardized sediment toxicity tests by regulatory agencies like Environment Canada [23] due to its close association with sediments. The way this species is exposed to contaminants depends on the type of contaminant and the specific exposure conditions. *H. azteca* typically inhabits the top 1–2 cm of sediment, where it builds burrows, but it also swims in the water column, maintaining constant contact with dissolved contaminants. As a detritivorous-grazer, it feeds on algae, diatoms, and decomposing organic material, and becomes contaminated mainly by ingesting periphyton or sediment particles. Compared to other aquatic species used in ecotoxicology, such as *C. riparius*, *H. azteca* is more sensitive to metals and less sensitive to organic compounds [44]. In its natural environment, *H. azteca* absorbs contaminants from the sediment, food (particulate phase), and overlying water (dissolved phase), with food being a significant source of contamination [54]. A limited number of studies have assessed *H. azteca*’s response to contaminants in both water and sediment [55,56,57,58], and these studies indicate that *H. azteca* primarily reacts to contaminants in the water column rather than those in the sediment or porewater.

### 3.9. Comparison of Heavy Metal Bioaccumulation in Hyalella azteca and Chironomus riparius with Controls

When examining the relationship between bioaccumulation and toxicity, it is essential to first consider the background levels of metal accumulation in amphipods exposed to natural (control) sediment [59]. The total heavy metal content measured in the tissues of *Hyalella azteca* and *Chironomus riparius* from before remediation, after remediation, SPM, and control sediments is presented in Table 3, Table 4, Table 5, Table 6 and Table 7. No statistically significant differences were found between the metal concentrations in the control and sediment samples from the study area. Sequential extraction data indicate that a significant proportion of metals are bound in stable forms within the sediment. Consequently, there is no substantial difference in metal concentrations between the test organisms exposed to the study area sediment and the controls. This conclusion is further supported by the survival and growth results, which show no significant difference in survival or growth due to the low levels of available heavy metals in the sediment’s available fraction. Thus, measuring metal concentrations in organisms can provide additional evidence to help identify contaminants potentially responsible for impairing benthic invertebrate communities [59,60].

### 3.10. Comparison of Results for Total Toxicity Tests and Bioaccumulation for Before Remediation, After Remediation, and SPM

In this study, standard 10- and 14-day toxicity tests were conducted using *Hyalella azteca* and *Chironomus riparius*, following Environment Canada protocols, to assess survival, growth, and metal bioaccumulation in sediment samples collected before remediation, after remediation, and from suspended particulate matter (SPM). The results indicated no significant toxic effects for either species across all treatments. Survival rates exceeded the minimum thresholds required for test validity, and relative growth rates did not differ significantly between test sediments and controls. Bioaccumulation data showed low metal uptake in both organisms, with no association to adverse biological effects. Comparatively, sediments collected before remediation, after remediation, and SPM all demonstrated similar non-toxic outcomes, despite higher metal concentrations detected in the SPM fraction.

## 4. Conclusions

This study with *Hyalella azteca* and *Chironomus riparius* highlights the effects of exposure to heavy metal-contaminated sediments from the study area (before remediation, after remediation, and SPM). The selected toxicity tests allowed us to integrate biological responses at different heavy metal concentrations. According to the statistical analysis, the survival and growth of *Hyalella azteca* and *Chironomus riparius* were not significantly different between sediment samples and controls. This research investigated the bioaccumulation of a mixture of Cr, Ni, Cu, Zn, As, Cd, and Pb in *Hyalella azteca* and *Chironomus riparius* in sediment from the study area and compared it to control sediments. This study investigated the total contents of heavy metals measured in the tissue of the studied organisms, *Hyalella azteca* and *Chironomus riparius,* before remediation, after remediation, SPM, and in the control samples. Statistically significant differences were not found between the contents of several metals in the control and the sediment sample from the study area. Sequential extraction data reveal the presence of a large proportion of metals bound in stable forms in the sediment of the study area. Thus, there is no significant difference in metal contents found in test organisms between the controls and sediment samples from the study area. This interpretation is confirmed by the survival and growth results. Low available heavy metal content determined in the available fraction resulted in no significant difference in survival and growth between sediment samples from the study area and controls.

## Figures and Tables

**Table 1:** Pearson and Spearman correlations (R) for heavy metals in *Chironomus riparius* and chemical fractions in sediment.

Extraction Steps	Cr	Ni	Cu	Zn	As	Cd	Pb
Exchangeable fraction (F1)	**−0.68** **	−0.51	**−0.72** **	**−0.60** *	−0.36	−0.15	0.17
Acid soluble fraction (F2)	−0.45	**−0.54** *	−0.36	−0.39	**−0.71** **	0.50	−0.50
Reducible fraction (F3)	−0.49	−0.36	−0.37	0.34	−0.39	0.29	−0.48
∑bioavailable (F1 + F2 + F3)	**−0.54** *	**−0.64** *	−0.36	−0.14	−0.39	−0.13	**−0.59** *
Oxidizable fraction (F4)	**−0.67** **	0.37	−0.51	−0.49	−0.13	−0.26	**−0.62** *
Residual fraction (F5)	0.23	0.46	−0.31	−0.27	0.42	0.29	**0.61** *
∑non-bioavailable (F4 + F5)	0.12	0.45	−0.51	**−0.58** *	0.39	0.01	−0.20
∑bioavailable + non-bioavailable (F1 + F2 + F3 + F4 + F5)	−0.29	**−0.67** **	−0.43	−0.47	−0.16	−0.12	−0.51
Total concentration	0.49	0.32	−0.21	0.26	**0.74** **	0.37	−0.07

* Correlation is significant at the 0.05 level (2-tailed). ** Correlation is significant at the 0.01 level (2-tailed). Significant values are bolded.

**Table 2:** Pearson and Spearman correlation (R) for heavy metals in *Hyalella azteca* and chemical fractions in sediment.

Extraction Steps	Cr	Ni	Cu	Zn	As	Cd	Pb
Exchangeable fraction (F1)	0.17	0.23	0.36	0.16	−0.30	−0.15	−0.04
Acid soluble fraction (F2)	**0.46** *	**0.46** *	**0.59** **	0.38	**0.54** **	0.28	0.10
Reducible fraction (F3)	**0.51** *	0.35	**0.62** **	0.10	**0.52** *	0.14	0.33
∑bioavailable (F1 + F2 + F3)	**0.47** *	**0.42** *	**0.66** **	0.29	**0.47** *	−0.07	0.22
Oxidizable fraction (F4)	0.11	0.10	0.06	0.35	0.38	−0.23	0.28
Residual fraction (F5)	0.14	0.19	0.02	−0.06	0.01	0.11	0.31
∑non-bioavailable (F4 + F5)	0.17	0.17	0.06	0.04	0.05	−0.22	0.34
∑bioavailable + non-bioavailable (F1 + F2 + F3 + F4 + F5)	0.38	**0.42** *	0.38	**0.45** *	0.38	−0.09	0.37
Total concentration	0.08	0.15	0.32	−0.15	−0.28	−0.23	0.28

** Correlation is significant at the 0.01 level (2-tailed). * Correlation is significant at the 0.05 level (2-tailed). Significant values are bolded.

**Table 3:** Comparison of bioaccumulation of heavy metals in *Hyalella azteca* with control for before remediation samples (µg/g).

Metal	Control	Station 1	Station 3	Station 4	Station 14
Cr	0.81	0.88	1.18	0.49	0.84
Ni	1.93	0.73	0.98	17.54	0.72
Cu	76.16	130.61	99.35	124.20	82.79
Zn	119.28	60.85	95.98	136.90	139.48
As	0.62	0.63	0.42	0.57	0.32
Cd	0.59	0.95	0.82	0.40	0.50
Pb	0.59	1.00	1.10	0.60	0.48

**Table 4:** Comparison of bioaccumulation of heavy metals in *Hyalella azteca* with control for after-remediation samples (µg/g).

Metal	Control	Station 1	Station 3	Station 4	Station 14
Cr	2.91	0.64	1.82	5.13	3.63
Ni	6.72	0.74	2.23	10.62	4.07
Cu	119.99	182.54	165.52	105.42	122.75
Zn	316.00	1176.38	317.08	178.68	114.69
As	1.50	0.97	0.72	0.73	0.69
Cd	1.08	1.78	1.63	1.23	1.82
Pb	1.95	0.812	0.94	0.72	1.37

**Table 5:** Comparison of bioaccumulation of heavy metals in *Hyalella azteca* with control for SPM samples (µg/g).

Metal	Control	Station 1	Station 3	Station 4	Station 14
Cr	1.19	9.00	1.71	0.66	0.82
Ni	0.682	2.73	1.65	2.13	0.45
Cu	87.25	254.79	184.00	165.21	129.41
Zn	97.45	130.87	66.74	75.31	63.58
As	0.81	0.48	0.67	0.55	0.46
Cd	0.56	2.01	0.92	2.09	1.01
Pb	0.14	0.31	0.42	0.18	0.35

**Table 6:** Comparison of bioaccumulation of heavy metals in *Chironomus riparius* with control for samples before remediation (µg/g).

Metal	Control	Station 1	Station 3	Station 14
Cr	1.31	2.56	1.63	2.62
Ni	1.66	1.73	1.78	2.52
Cu	31.39	50.00	35.94	69.28
Zn	133.78	260.48	279.20	234.63
As	0.38	0.08	0.19	0.47
Cd	0.79	1.16	1.52	2.47
Pb	0.82	1.72	0.85	1.74

**Table 7:** Comparison of bioaccumulation of heavy metals in *Chironomus riparius* with control for after-remediation samples (µg/g).

Metal	Control	Station 1	Station 3	Station 4	Station 14
Cr	1.41	1.41	1.12	1.02	1.33
Ni	2.11	2.11	1.36	0.74	0.99
Cu	23.37	23.37	61.69	35.31	37.42
Zn	115.83	115.83	208.44	108.26	168.28
As	0.40	0.40	0.13	0.15	0.17
Cd	0.68	0.68	2.31	1.76	1.83
Pb	0.77	0.77	0.78	0.48	0.65

## Data Availability

Data are available upon request.

## Supplementary Materials

The following supporting information can be downloaded at: https://www.mdpi.com/article/10.3390/toxics13060432/s1, Table S1: Physical characteristics of sediment samples before remediation; Table S2: Physical characteristics of sediment samples after the remediation and SPMs; Table S3: Total concentration of heavy metals in sediment samples (mg/kg) (The bold and highlighted numbers are the concentrations of elements exceeding the OEL and PEL respectively); Table S4: Total concentration of selected heavy metals after the resuspension test (mg/kg) (The bold and highlighted numbers are the concentrations of elements exceeding the OEL and PEL respectively); Table S5: Total concentration of selected heavy metals in SPMs (mg/kg). (The bold and highlighted numbers are the concentrations of elements exceeding the OEL and PEL respectively); Figure S1: Comparison of the survival percentage of *Hyalella azteca* between the controls of the different tests (batch) and after remediation samples at various stations; Figure S2: Comparison of the RGR percentage of *Hyalella azteca* between the controls of the different tests (batch) of after remediation samples at various stations; Figure S3: Comparison of the survival percentage of *Hyalella azteca* between the controls and SPM samples at various stations; Figure S4: Comparison of the RGR percentage of *Hyalella azteca* between the controls and SPM samples at various stations; Figure S5: Comparison of the survival percentage of *Chironomus riparius* between the controls and after remediation samples at various stations; Figure S6: Comparison of the RGR percentage of *Chironomus riparius* between the controls and after remediation samples at various stations.
