# Supplementary material for: Metal Exposure, Bioaccumulation, and Toxicity Assessment in Sediments from the St. Lawrence River Before and After Remediation Using a Resuspension Technique
Source: Toxics. 2025 May 25;13(6):432. doi: 10.3390/toxics13060432 (PMC12197031; doi:10.3390/toxics13060432)
Supplement: Supplementary file 1 [file toxics-13-00432-s001.zip › toxics-3629450-supplementary.pdf]

Supplementary Material

Table S1. Physical characteristics of sediment samples before remediation

St. No	LOI%	D_{50} (µm)	Clay (%)	Silt (%)	Sand (%)	Pebble (%)
1	9.2	325	1.36	19.02	71.6	11.9
3	14.3	279	1.22	19.7	75.6	5.2
4	14.9	63.4	5.06	49.5	45.2	0.22
6	5.9	51.5	10.5	50.3	37.7	4.2
8	11.1	25.8	12.2	66.3	21.4	
9	15.6	73.5	7.44	46.9	45.6	
11	10.8	14.6	17.5	67.2	15.3	
14	9.3	7.12	47.6	42.8	9.5	

Table S2. Physical characteristics of sediment samples after the remediation and SPMs.

	Sediment -after the test						SPM				
St. No.	LOI%	D_{50} (µm)	Clay (%)	Silt (%)	Sand (%)	Pebble (%)	LOI%	D_{50} (µm)	Clay (%)	Silt (%)	Sand (%)
1	11.7	96.0	9.85	33.7	47.0	9.3	9.6	5.28	44.6	52.2	3.11
3	11.6	42.4	12.63	41.9	41.6	5.7	13.5	1.36	61.5	37.8	0.54
4	18.5	78.7	6.28	41.1	47.0	11.2	13.0	9.66	7.14	91.8	1.09
6	5.9	31.3	10.01	47.1	42.6	0.22	9.8	4.56	96.9	3.05	
8	15.1	51.8	12.76	38.9	47.4	2.67	11.3	0.24	72.0	25.9	2.05
9	14.2	26.3	5.45	58.7	35.5	0.39	13.4	0.17	99.6	0.39	
11	11.5	28.6	25.01	40.8	32.7	4.1	10.7	0.28	62.7	35.9	1.38
14	11.5	7.2	36.13	41.8	21.8	0.48	9.99	0.23	81.1	18.7	0.21

Table S3. Total concentration of heavy metals in sediment samples (mg/kg) (The bold and highlighted numbers are the concentrations of elements exceeding the OEL and PEL respectively).

St. No	Cr	Ni	Cu	Zn	As	Cd	Pb
1	55.9	29.2	**450**	**243**	7.21	0.75	**63.6**
3	**61.1**	32.6	**255**	**248**	**8.6**	0.75	**56.4**
4	**66.9**	35.61	**164**	**304**	7.68	0.99	49.3
6	41.6	28.1	34.8	138	5.49	0.47	22.3
8	**68.6**	38.1	118.4	**247**	**9.93**	0.77	59.4
9	**78.1**	40.8	126.1	**229**	8.65	0.88	54.5
11	**69.2**	35.7	66.6	**223**	6.65	0.75	31.1
14	**74.7**	42.3	**127**	**215**	**15.52**	0.77	32.7

Table S4. Total concentration of selected heavy metals after the resuspension test (mg/kg) (The bold and highlighted numbers are the concentrations of elements exceeding the OEL and PEL respectively).

St. No	Cr	Ni	Cu	Zn	As	Cd	Pb
1	43.4	24.2	252	**200**	5.3	0.63	45.9
3	**60.3**	32.4	**137**	**233**	7.7	0.70	**55.7**
4	**66.7**	34.5	**137**	**329**	5.7	1.12	51.4
6	31.8	20.3	31.4	113.0	3.5	0.37	15.8
8	53.5	34.8	55.02	**229**	5.5	0.90	30.9
9	**77.3**	41.0	**103**	**255**	7.1	0.80	35.4
11	**57.3**	34.5	47.8	**199**	5.2	0.74	28.3
14	**69.1**	42.5	**72.4**	**229**	7.15	0.76	36.5

Table S5. Total concentration of selected heavy metals in SPMs (mg/kg). (The bold and highlighted numbers are the concentrations of elements exceeding the OEL and PEL respectively).

St. No	Cr	Ni	Cu	Zn	As	Cd	Pb
1	**84.2**	**52.3**	301.5	358.1	6.08	0.81	48.5
3	**78.3**	**78.3**	209.0	314.9	6.95	0.42	47.1
4	**76.6**	44.8	**195.7**	**316.6**	4.4	0.92	37.5
6	**79.6**	**47.9**	**164.9**	**288.4**	4.4	0.42	29.9
8	**77.3**	**47.5**	**140.8**	370.6	5.3	0.47	31.1
9	102.7	**56.7**	**163.7**	346.3	7.9	0.70	35.9
11	**82.4**	**82.4**	**158.9**	311.9	5.1	0.49	29.0
14	**80.5**	**47.1**	**141.5**	**298.4**	4.9	0.45	31.9

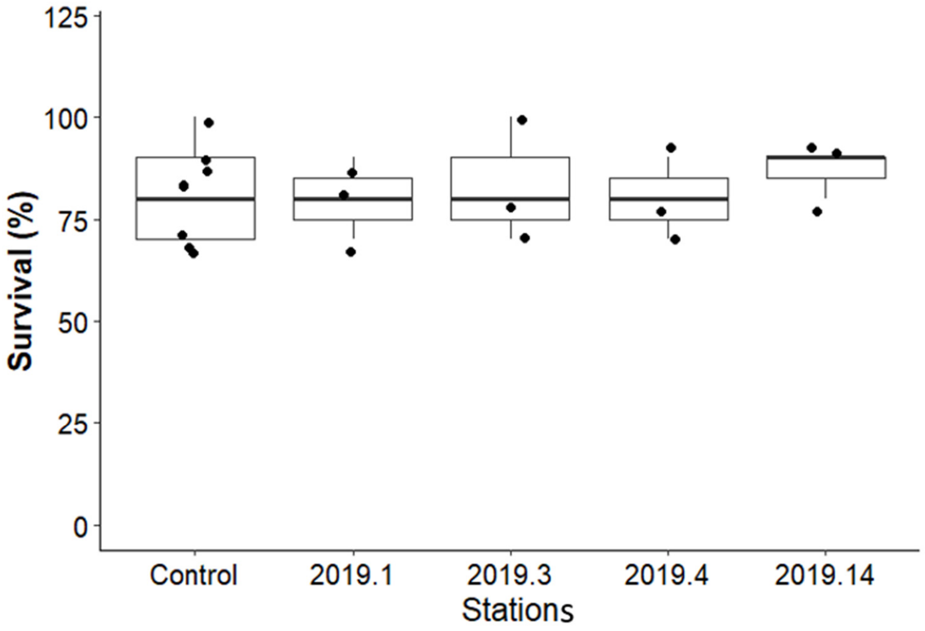

Figure S1. Comparison of the survival percentage of *Hyalella azteca* between the controls of the different tests (batch) and after remediation samples at various stations.

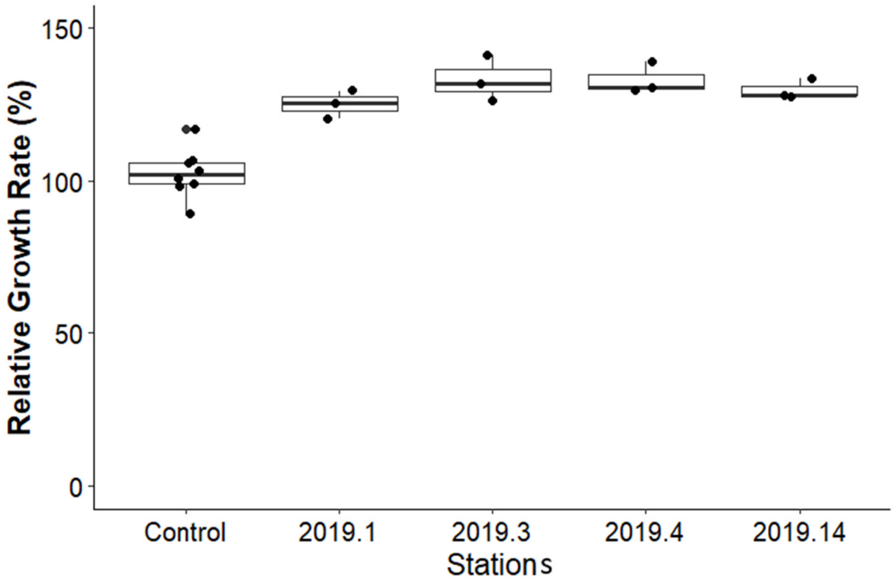

Figure S2. Comparison of the RGR percentage of *Hyalella azteca* between the controls of the different tests (batch) of after remediation samples at various stations.

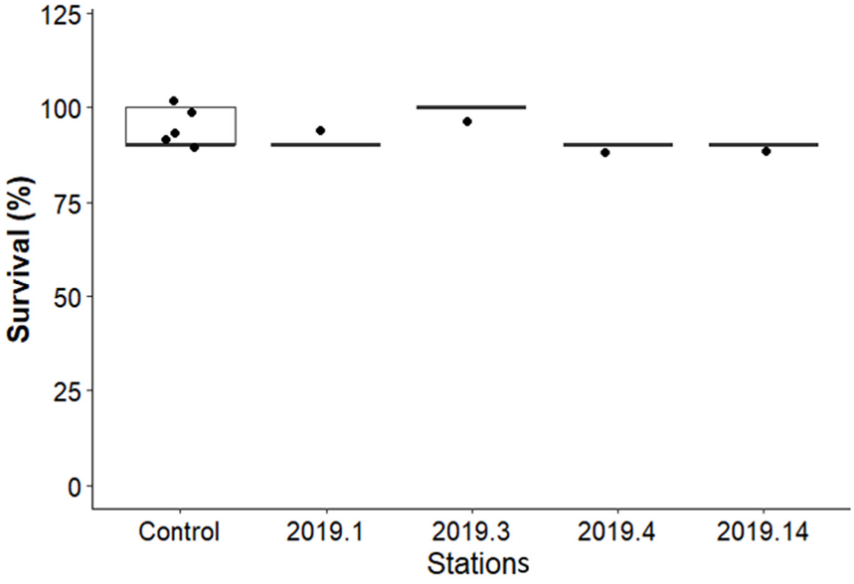

.

Figure S3. Comparison of the survival percentage of *Hyalella azteca* between the controls and SPM samples at various stations.

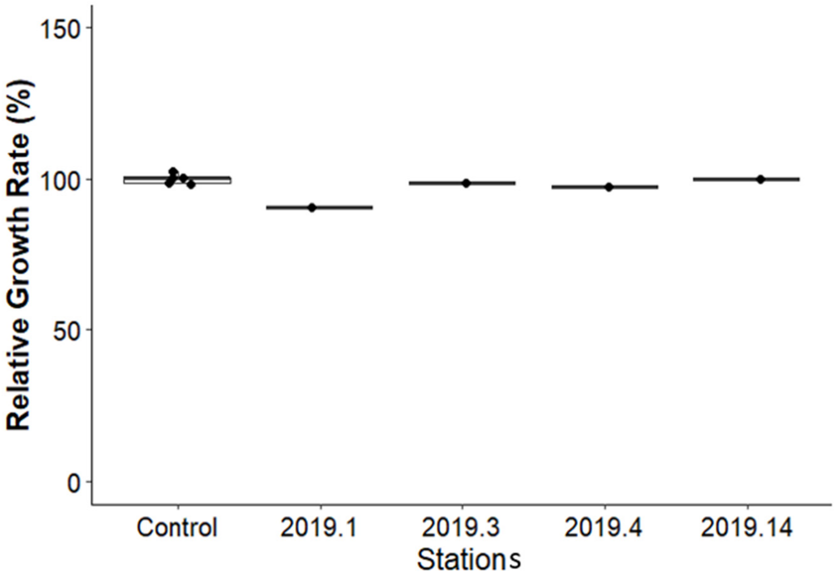

Figure S4. Comparison of the RGR percentage of *Hyalella azteca* between the controls and SPM samples at various stations.

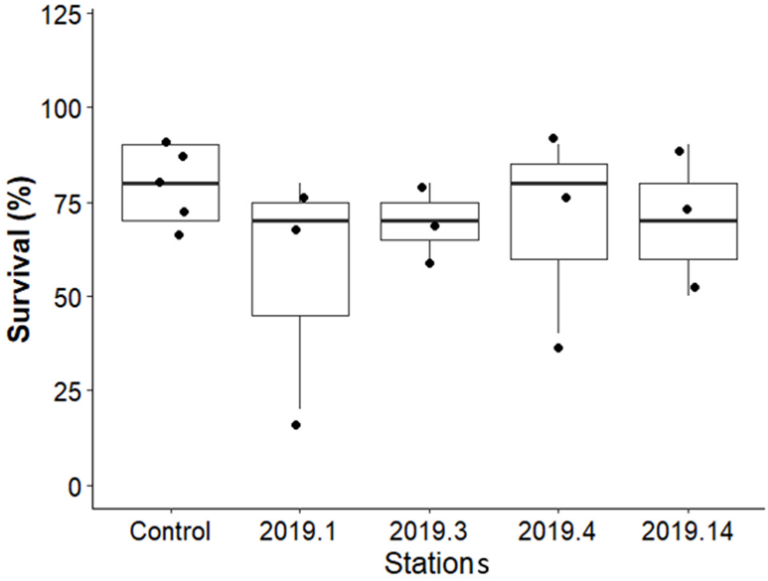

Figure S5. Comparison of the survival percentage of *Chironomus riparius* between the controls and after remediation samples at various stations.

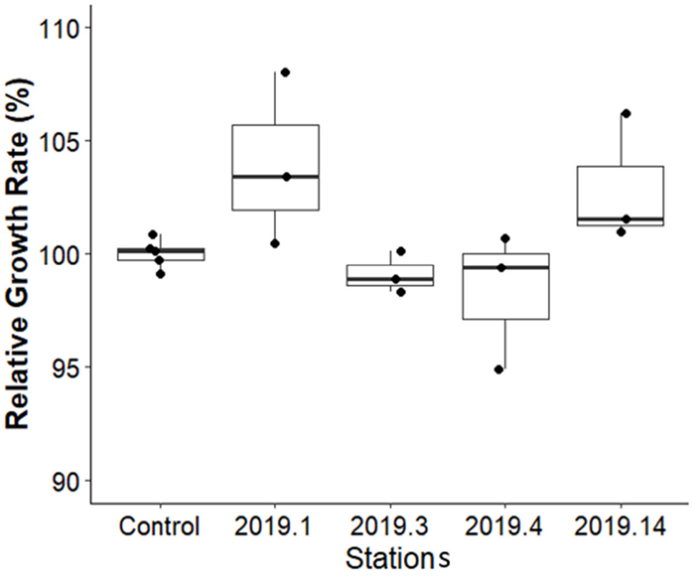

Figure S6. Comparison of the RGR percentage of *Chironomus riparius* between the controls and after remediation samples at various stations.
